# Extra-Limites

**Published:** 1834-01-01

**Authors:** 


### EXTRA-LIMITES.

### Observations upon the Critique on Mr. Carmichael's Paper, &c. &c.

Sir,—In your last Number, there is a critique on a Paper inserted by me in the Dublin Medical Journal for this month, "on Inflammatory Affections of the Brain and its Membranes," which demands from me some observations, not only because, in the critique in question, some misapprehensions and oversights require correction, but that a further discussion of the subject included under the title of my paper, may lead to more defined and precise notions respecting the nature and appropriate mode of treatment of a numerous and important class of diseases.

Upon the first case detailed, one in which the inflammatory affection of the brain and its membranes was indicated merely by a pain in the ear, shooting from thence along the side of the head, the reviewer indulges in the following animadversions; which, on a consideration of the facts, he will scarcely consider himself justified in making.

"We must confess that, great as is our respect for the talents and attainments of the gentleman concerned, we do not approve of the treatment pursued in the preceding case. A plethoric person has violent pain in the side of the head, which has lasted for some time, and been aggravated by imprudences, and is attended with loaded tongue and quick pulse. He is relieved by leeches and cathartics. We would rather have bled the patient than leeched him ; but let that pass. Two days afterwards he is again found suffering from pain, and he has sickness of stomach. Under these circumstances, he is orded three grains of calomel and ten of Dover's powder, with cathartics and a blister. Why was opium given? Under such circumstances, bleeding or cupping would surely have been preferable to the blister, and an active system of purgation with calomel, in our humble opinion, have been the most appropriate remedies. He is now, however, put on mercury and improves a little, when he is ordered bark, with hyosciamus and camphor. Again, after this, narcotics are given. He dies with purulent effusion between the membranes. We repeat, that we think the patient would have a better chance of recovery if he had been more actively treated in the first instance, and more antiphlogistically throughout. We have noticed the case with the view of cautioning practitioners, for, unfortunately, we have seen others not very dissimilar."

A reviewer elevates himself into the dignified situation of a judge, and he, of all human arbiters, in detailing the grounds upon which he forms his judgment, ought to be cautious not to suppress important points of evidence, or give a false colouring to any circumstances of the case. This, observation will, I think, be sufficiently substantiated, in the case under consideration, by comparing the reviewer's statement with the original Essay. The reviewer says—" Mr. C. considered the case one of inflammation, ordered sixteen leeches near the ear, and cathartic medicine, containing tartarized antimony, to be taken every hour. In the evening the patient was better, and *Mr. C. did not see him again till the 20th.*" Would not any one conclude, from the above passage in Italics, that my postponing to see my patient till the 20th was my own voluntary act, arising from a belief in his safety? In candour and fairness, my reviewer ought not to have omitted the the following passage, which appears in the original statement:—" I saw him again in the evening (18th March); the leeches had caused an *unusually profuse discharge of blood,* and the medicine had affected his bowels ; his countenance was composed, and he said that *he felt so perfectly relieved from pain, from what had been done, that he would not again trouble me to visit him.*"

If, however, the reviewer had given this passage, he could scarcely afterwards have added, in commenting on the futility of leeches in such a case—" we would rather have bled him—but let that pass." An omission of such a hit as this would not, it seems, from the context of the critique, have answered our reviewer's purpose.

That which marks the difference and grades of intellect amongst individuals of our profession, is the power or tact of tracing symptoms to their sources in the commencement of disease; and this at times is so difficult a task, that men of the most powerful minds and of the most extended experience are not ashamed to acknowledge, that they have often erred in their judgment, until the progress of the malady unfolded clearer views and a more certain diagnosis. When once a disease is fully developed, the path to be pursued is plain, and easily followed by even the dullest and most ignorant pretender to medical science; therefore my reviewer has little to boast of, in the display of his superior judgment over the author he criticizes, when a post-mortem examination had demonstrated to him the true nature of the disease; and then to proclaim with a commanding air the identical treatment, which was plainly the object the author had insisted upon in his paper, seems, to say the least of, rather bombastical. But would this gentleman, who finds it so easy a task to lay down the practice that he conceives ought to have been pursued after the case has terminated, and dissection has left no doubt of the true seat and nature of the disease, have found no difficulty in deciding upon the appropriate line of treatment, if he had seen the patient at an earlier stage? Has he not observed most painful neuralgic affections of the ear, temple, and side of the head and face, occurring in dyspeptic persons, which often simulate an inflammatory attack? and might not all his general bloodlettings, blisters, and strong mercurial purgatives increase, instead of diminishing the painful symptoms of such a malady? I did not consider the case to be one of neuralgia; but, if I had so considered it, I should not be ashamed or afraid to state a mistake, which, in this instance, was very likely to occur, as the symptoms more strongly resembled a neuralgic than an inflammatory affection.

The patient had been for years a very dyspeptic person, therefore the more liable to neuralgia. My treatment was cautious, as I deemed it prudent to feel my way. Leeches were preferred to general blood-letting, because there was not decided evidence that the disease was inflammatory; but the leeches and antimonial purgatives relieved the pain on the first day so completely, that the patient himself intimated that my attendance was no longer requisite. The relief afforded strong grounds that I was right in the treatment I pursued. The pain recurred, but with far less violence, and I determined to mercurialize the system—a measure calculated to benefit the patient in either case, whether neuralgic or inflammatory; and Dover's powders were given *once* conjoined with calomel, with a view to relieve pain. It is unnecessary to hint, that opium may be given in inflammation, after the force of the circulation is reduced, with safety and the best effects.—Witness Dr. Armstrong's excellent practice of exhibiting a large opiate in peritonitis after bleeding. This is my answer to the question, "Why was opium given?" The pulse during this treatment fell on the 21st to 80, and the pain was so far relieved, that I was a second time released from farther attendance by the desire of the patient.

Although the pain was at times lancinating, shooting along the temples and side of the head, partaking very much the character of neuralgia, yet from the first I considered it to be connected with inflammation. What indications in the gross determined my judgment I cannot now distinctly detail. I have every day witnessed more severe pain and equal quickness of pulse attendant upon neuralgic affections; but in that arising from inflammation, though more severe at one time than another, and even this encrease returning at regular periods, yet *some pain* is always present. The reverse we know to be the case in neuralgic affections, the patient between the paroxysms being *totally free* from pain. A practitioner draws his conclusions from the totality of the symptoms amongst which the expression of the countenance is of the utmost importance. In this instance, as well as in others where neuralgial inflammation existed, I have observed the expression of the eye to be often dead and languid; but it is in vain to attempt by words to convey an adequate notion of the peculiarity of expression which seems to attach itself to each individual disease. This can only be learned by the practitioner's tact and talent for observation. Thus a very different mode of expression attends phthisis, hepatitis, peritonitis, pleuritis, meningitis, and various other affections. The expression of the eyes in tetanus announces to the first glance of the practitioner the nature of the disease, even before the patient feels his jaws begin to stiffen; and yet it is impossible to convey by words any notion of the peculiarity of this expression. I fear that by some I shall be esteemed garrulous in making these observations, but many will perhaps coincide with me, that a practitioner of experience will anticipate justly in his mind the nature of the organic disease with which a patient is affected by the very expression of his countenance, before he hears a word on the symptoms or history of the case submitted to his judgment; and it was the exercise of this power of morbid physiognomy which principally induced me to consider the disease in the present instance to be of an inflammatory nature. Another circumstance concerning this interesting and important case is worthy of remark. Although the pain was referred chiefly to the ear, yet no signs of inflammation of that organ was discernible. An analogous instance of a patient dying of meningitis, (notwithstanding the strongest antiphlogistic measures,) in whom the disease was indicated by violent pain in the eye, and yet that organ was found free from inflammation, is referred to in my paper.

These two instances alone are sufficient to put us on our guard against inflammation of the brain or its membranes, whenever we meet with acute attacks of pain in the ear attended with pyrexia, in which neither of these organs indicate the other usual signs of inflammation.

One interesting case well sifted and analyzed is more instructive than a hundred superficially detailed; I therefore do not regret that the animadversions of my reviewer have excited this discussion, as the observations of the practitioner who witnessed the case are necessarily more entitled to confidence than those of any other individual, unless indeed he happen to be a reviewer; and I am not disposed to cavil at any man's privileges.

As a proof of my obsequious submission to prerogative, I shall abruptly terminate the discussion, although my reviewer has left me ample opportunities of commenting on a fastidiousness that nothing can please. Even my second case though beyond measure successful, excites his displeasure as much as the first. My third case "presents nothing of interest,"—my fourth, though acknowledged to be interesting, is "sadly diffuse in narration." In the fifth I am accused of being a regular Emerald Islander; and the sixth case, though given by the reviewer in full, and detailed for the purpose "of shewing the general character of the treatment recommended by Mr. C., is such as possibly occurs to all surgeons or physicians engaged in practice."

Our reviewer after *fibbing* me in this pleasant kind of way, good humouredly closes the set-to by patting me gently on my organ of self-esteem, and observing "we trust no apology is necessary to Mr. C. for having criticised his opinions freely—for Mr. C.'s talents we entertain a high respect, &c." The oiliness of this panacea is well adapted for the embrocation of bruises far sorer than any I have suffered on the present occasion. But I cannot refrain from thinking that both gauntlet and plaister were supplied from this side of the channel. I know that I am not without one or two kind and goodnatured friends, who would like to benefit my health even at the expense of my practice. They have been observed of late galloping about town, and flourishing in the faces of people on all apposite occasions, a large blue book entitled the 38th number of the Medico-Chirurgical Review, but let them write and gallop as much as they please, they will not find it quite so easy a task as they fondly imagine to gallop over or write me down. If a man honestly flourishes, I enjoy his prosperity; but I tell the pretender who flourishes by foul means, that his flourishing is in vain, he will not flourish long.

### NOTE BY THE EDITOR.

Dr. Johnson has given insertion to Mr. Carmichael's Reclamation, in order that he may set himself right on all points where he is, or thinks himself aggrieved by the Reviewer. But Dr. J. can assure Mr. Carmichael that his Reviewer is no secret enemy near himself—nor an enemy at all. The Reviewer never saw Mr. C. in his life—and never had his foot on Irish soil. He is a Gentleman attached to a large public Hospital in this Metropolis—has had ample experience in surgical cases—and Dr. J. will, at any time, disclose his name, privately, to Mr. Carmichael. Dr. J. has much pleasure in stating this fact, in order to relieve Mr. Carmichael's mind from the idea of having secret enemies in Dublin, who write against him in the "large blue book." That book never yet inserted an *anonymous critique* on any medical or surgical work; and never, since its first existence, published a critique of any kind from the pen of a member of the profession in Dublin—or, to the best of Dr. Johnson's memory, from the pen of any man in the Emerald Isle. It is very true that Dr. J. cannot, from the extent of his other avocations, write the whole of a Journal, which is entirely REVIEW; but he writes more of it than some people give him credit for; and he employs none for the purpose of assisting him, but those on whose talents and integrity he can depend. Had this not been the case, THE MEDICO-CHIRURGICAL REVIEW would not have attained its present circulation, and it would not have had the *costly* honour of being the only Medical Journal that was ever *re-published* on a foreign soil. Dr. J. says *costly* honour, since that re-publication, though it does not diminish the circulation at home, yet it supplies more than a thousand subscribers beyond the Atlantic.

---

N. B. In consequence of the press of matter this Quarter, the Bibliographical Record is unavoidably postponed till the next Number.

